# Introductory Address

**Published:** 1863-04

**Authors:** Anthony Hockley


INTRODUCTORY ADDRESS—*Delivered on Wednesday, October 3, 1862, at the Opening of the Fourth Session of the Metropolitan School of Dental Science. By Anthony Hockley, M.C.D.E. Lecture on Dental Mechanics to the School.*

Gentlemen:—There are occasions when we are called upon to undertake duties which we do not feel we can adequately fulfill. Such, I assure you, was the case with myself when I yielded to the request of my colleagues, and consented to deliver the introductory address on the opening of the present Session. I feel myself wanting in the fluency and aptness of language to which you have been accustomed to listen, and I must crave your indulgence and ask those I see around me to accept in a friendly rather than a critical spirit what may fall from me this evening.

If much that is novel can not be imparted to an Introductory Address, it should not be forgotten that our audience to a certain extent changes annually—that is, the elder students pass away into practice, others are entering the career of student life, and the same words of encouragement and advice are applicable to them as was formerly pronounced to their predecessors.

Those of my colleagues who have preceded me on these occasions dwelt upon the scientific and surgical history of the Dental Profession, and I shall therefore speak more particularly on the progress which has been made in Mechanical Denistry; first, because it has been less fully dilated upon; and secondly, because it is the subject upon which I feel most competent to address you.

The teeth form so prominent a feature in the human countenance, and are so essential to the enjoyment of health, that in every country where the people have made any considerable progress in the arts and sciences of civilized life, the care of these organs has become sooner or later a special branch of medical practice. In Egypt, where the principle of the subdivision of labor was carried to excess, and where every art and profession was handed down from father to son, and every calling was hereditary, the practice of Dentistry appears to have been in the hands of a special class of practitioners. How far they were acquainted with the diseases of the teeth it is useless to enquire. It seems that they made some attempts to supply the loss of these organs by artificial substitutes, for in the sarcophagi of Egypt artificial teeth made of sycamore wood have been fonnd. Moreover, mummies have been discovered in which teeth had been filled with gold. It has been questioned by some whether the operation was performed during the life of the individual, or whether it formed part of the embalming process, and that the gold was inserted in the teeth after death, for the purpose of preserving those organs from destruction.

The Greek and Roman writers (with the exception of Galen and Ætius) were but imperfectly acquainted with the physiology of the teeth. What progress these nations had made in Dental Surgery, may be gathered from a circumstance mentioned by Eristratus, that an instrument made of lead for extracting teeth was suspended in the temple of Apollo, to indicate that teeth should only be drawn when they had become sufficiently loose to yield to so feeble an instrument. The Roman author, Galen, has left a correct account of the number and form of the teeth, and describes several operations; among others, stopping teeth, which was resorted to to prevent them from breaking from the pressure of the instrument when extracted. Neither he nor Celsus make any allusion to artificial teeth, although the latter speaks of fixing loose teeth by means of gold or silver wire, a plan adopted 500 years previously by Hippocrates. It is certain, however, from the remarks in the poems and satires of the Roman writers, that the ladies of the Imperial city were acquainted with the use of artificial teeth, and had recourse to them to aid the charms of beauty. Martial, in one of his satires, addressing himself to Lelius, says: "You are not ashamed to purchase teeth and hair." He also refers to the toothless mouth of Egle being repaired with bone and ivory, and speaks of Galla removing her artificial teeth during the night. He mentions the name of Cascillius as a person who was in the habit of fastening as well as extracting teeth.

Little is to be learned concerning dentistry during the dark period of the middle ages when ignorance and barbarism spread itself over Europe. With the exception of Albucasis, an Arabian physician who lived in the twelfth century, and who was the first to mention that the teeth may be replaced by artificial ones made from the bones of an ox, it is in vain that we seek for any information concerning the progress of Mechanical Dentistry until the middle of the sixteenth century.

In the works of Ambrose Paré, who lived between 1509 and 1590, and was surgeon to Henry II and Charles IX of France, there are several references to the diseases of the teeth and their treatment. In his celebrated work on Surgery, an English translation of which was published in 1634, Paré devotes several chapters to the subject, and is the first to describe the operation of transplanting teeth. With respect to artificial teeth, he says: "Teeth artificially made of Bone or Ivorie may be put in the place of those that are wanting, and they must be joined one fast within the other, and also so fastened unto the natural teeth adjoining that are whole; and this must be chiefly done with a thread of gold or silver, or for want of either, with a common thred of silk or flax, as it is declared at large by *Hippocrates*, and also described in the Figure following." The "figure" referred to consists of a rude representation of artificial teeth and the wires by which they were attached. It is curious to observe how little they resemble in any way the form of the natural teeth, so that their presence must have been immediately detected, and in the present day we should regard them as really a greater disfigurement than the absence of the natural organs. Guillemeau, who was a pupil of Paré's, recommended artificial teeth to be formed of a paste composed of white wax melted with a small portion of gum elemi, adding to it powdered mastic, white coral, and pearls. With this paste he informs us artificial teeth may be made which will not discolor, and he adds that the composition will also be found useful in stopping teeth. Amongst other matters, Paré refers to the subject of artificial palates, but they had been described by previous writers. The earliest plan adopted to remedy these defects was to stop up the apertures with wax or sponge. Alexander Petronius, in a work published fourteen years before Paré, is the first to mention the use of a gold plate for this purpose. The obturator described by the great French surgeon consisted of a gold or silver plate about the thickness of a French crown, and a little larger than the opening in the palate. It was of a concave form towards the cavity of the mouth, and had a piece of sponge attached to its upper surface, which, as it become swollen by absorbing the moisture of the part, held up the obturator.

We learn from this inquiry that by the middle of the sixteenth century the use of artificial teeth was known, but that they were seldom employed, while with regard to operations on the teeth, extraction was the one principally performed, and that with instruments of the rudest possible description. Stopping teeth was referred to, but was rarely adopted as a remedy for caries.

With regard to the surgical department of the profession at the period when Ambrose Paré wrote, we find that the old pelican continued to be used for the extraction of teeth, until the time of Garengeot, who modified it to a certain extent, and is generally spoken of as the inventor of the key instrument. The operation of extracting was at this time no trifling one: and, indeed, when we contemplate the ill-constructed instruments which were used for the purpose, it is little to be wondered at. The hold which these instruments obtained of the teeth was most imperfect, and the force used was applied at a mechanical disadvantage. To look at these instruments, and to read Paré's description of the mode in which they are to be used, is sufficient to justify those surgeons who questioned the propriety of tooth-extraction, and forms a reasonable excuse for the general disinclination which the educated practitioner felt to operate upon these organs. The preliminary operation of lancing the gums was carried to an extent which rendered it almost as painful as the actual extraction is at the present day. The patient was seated on the ground or on a low stool, and his head was fixed between the legs of the operator. Then commenced the process of lancing the gums. What this meant may be gathered from the following: "Then the tooth-drawer shall deeply sacrifice about the tooth, separating the gums therefrom, and then, if spoiled, as it were, of the wall of the gum, it grows loose" —mark the word "*loose*," gentlemen, and then consider what a pleasant operation this said lancing of the gums must have been—"it must be shaken and thurst out, by forcing in with the three pointed Levatory." Happy was the patient if this terminated the operation; but woe betide him if, as the writer says, "it stick too fast and will not stir at all," for "then must the tooth be taken hold of with some of these toothed *forcipes;* now one, then another, as the greatness, figure, and site seem to require." Truly it was desirable that the opeartor should be somewhat expert in the use of the "toothed Mullets," as they were termed, for it is admitted that "unless one know readily and cunningly how to use them, he can scarce so carry himself but that he will force out three teeth at once," whilst to crown the operation, "that which caused the pain was left oft-times untoucht." The records which remain in the surgical works of the period of fractured jaws, abscesses, and violent hæmorrhage, testify too strongly to the truth of the above account of what tooth-drawing was in the 16th century.

Up to a comparatively recent period the key-instrument was the one in vogue, and similar injurious results to the one described too often occurred to afford any doubt of the wide scope which existed for improved methods of operating.

We may then regard this as the first step in the foundation of Dentistry, and it will be observed that at this time it was in the hands of medical men. The observations on the teeth contained in surgical works were exceedingly superficial, and were only inserted that the writers should not appear to ignore anything which might be considered as belonging to their subject.

From this time little or no progress was made until 1700, when Fauchard, a French surgeon, turned his attention to dental practice, and confined himself exclusively to the treatment of the teeth and their diceases. To Fauchard, who has been justly termed the father of Dental Science, we are indebted for the first complete treatise on Surgical and Mechanical Dentistry. This work, entitled, "Le Chirurgien Dentiste," was published at Paris in 1728. Up to this period only two special treatises on the teeth had been published, one by Urbain Hemand in 1582, upon the Anatomy and Diseases of the Teeth, containing principally the opinions of the Greek and Roman writers on the subject; the other, "A Dissertation on the Teeth," by B. Martin, in 1679, in which he describes the character of the teeth, and treats of their diseases, but does not speak of the operations relating thereto.

The work of Fauchard marks an important era in the history and progress of Dental Science, and is far in advance of any publication which had preceded it: in fact, it may be said to have been the first attempt to give a full and connected account of the diseases of the teeth, and their treatment, and the means which were then employed for making and fixing artificial substitutes. The work is illustrated by numerous engravings, descriptive of the anatomy of the teeth, surgical operations, the various implements used in the manufacture of artificial teeth. For the latter purpose he recommends natural teeth, sea-horse and walrus teeth, also some of the long bones of the ox, boiled and bleached in lime, as the substances to be employed. The difficulties which the dentist had to encounter were great; and it seems strange that better means were not used to overcome them. No impression of the mouth was taken, and in fitting pieces to the mouth, the workman trusted to his eye alone, and to such measurements as he could obtain of the shape and form of the mouth. This writer gives a minute description of his invention for fixing an entire upper piece, which, at that time, some dentists were in the habit of securing by means of gold wire passing through the gums in the manner of ear-rings; or the piece was held in its position by flat pieces of whalebone tied by thread to the teeth of the lower jaw. The improvement referred to consisted of two narrow strips of gold, one passing behind and the other in front of the lower teeth, the two pieces being connected by small pieces of flattened wire passing over the molar or such other teeth as remained. On the outer bar, and corresponding to where the swivels are now placed, a hole was drilled for fixing the springs. These springs, which were made of whalebone, strengthened with flat pieces of steel, were inserted into the upper piece by rivets.

Another ingenious invention of Fauchard's was a process of enamelling gold. A thin strip of the metal having been fashioned into the form of the outer surface of the teeth was then sent to the enameller, who covered the outer surface with a coating of enamel, colored to resemble the natural teeth and gums, or, as Fauchard adds, "to suit the fancy of the wearer." The gold thus prepared was then fastened on to the outer surface of a piece of bone previously fitted to the gum, and adapted to the contour of the mouth. This may fairly be considered as the first step toward the introduction of mineral teeth.

As a profession, dentistry was at this time at an extremely low ebb, being chiefly in the hands of barbers and itinerant operators. The regular surgeon avoided, as far as possible, all interference with operations on the teeth. Pierre Dionis, the professor of anatomy at Paris, in his lectures on surgical operations, published in 1707, declaims against these unqualified practitioners; yet, at the same time, he recommends the surgeon never to attempt the extraction of teeth, lest it should render his hand unsteady, and unfit him for the performance of other operations. Another reason he assigns is, lest the surgeon should be thought a charlatan, and be classed with a body of men who used their talents to deceive the public. He admits, however, that the surgeon should be acquainted with the theory, if not with the practice, of dental operations.

The next step in advance was made in 1757, when Bourdet suggested that a model of the mouth should be made in wax, and the artificial teeth adapted to the model. This was, of course, a much more convenient method of working; but, at the same time, the model must have been a very imperfect substitute for the plaster models of the present day. The material employed consisted of gold, bone, and natural teeth; and when the former material was used, the model was given to the goldsmith for the purpose of constructing a small plate or cup of metal, into which the natural tooth was inserted, and secured by a small piece of wire passing through the tooth and plate; holes were drilled in the plate for the purpose of securing it to the adjoining teeth. This plan, which is spoken of as an important invention, and may be considered as the first attempt to fix a gold plate, was chiefly applied in cases where there was absorption of the gums. Previous to fixing the tooth as above described, the gold plate, or cup, was sent to the enameller's to be coated with enamel, in imitation of the defective gums, thus requiring no less than three different workmen to complete the piece. Bourdet was also the first to insert natural teeth into bone sockets. The plan adopted by this ingenious inventor was to drill holes in the bone which had been previously fitted to the gum, and into these holes to insert the teeth with their fangs, fixing them in position by small pins passing through the bone and the teeth. He also fitted teeth upon the plan of devetailing them to the bone, and the drawings representing the teeth thus prepared, resemble in form the vulcanite teeth of the present day.

It was sometime after this that the plan of taking an impression of the mouth in wax was introduced. I am unable to say to whom we are indebted for this simple but important improvement; but certain it is that the obtaining of a correct model must have greatly facilitated the labors of the workman, who was previously in the habit of working to the mouth itself, the surface of the gums being covered with color; and the bone which was to form the artificial piece was fitted to them in the same manner as is now done to the plaster model.

Towards the latter part of the last century, two important inventions were introduced, which commenced a new era in Dental Mechanics—I refer to the introduction of gold plates, and the use of silicious compounds in the manufacture of mineral teeth. Talma, the father of the great French tragedian, has the credit of being the first to introduce gold plates into this country. In 1774, Duchateau, a chemist of St. Germain en Laye, first conceived the idea of manufacturing mineral teeth; but the invention was brought to greater perfection by Dubois Dechemant, who claimed it as his discovery. The dispute as to the priority of the invention became the subject of a law-suit, which was decided in favor of Dechemant. So important was the invention of mineral teeth considered, that the Medical Society of Paris offered a medal for the best essay on the materials for coloring and enamelling the teeth, and for the best means of adjusting them to the mouth without injury to the remaining teeth.

After the appearance of Dechemant's invention, which was secured to him by letters patent in England and France, several years elapsed before any further progress was made in the manufacture of mineral teeth. About 1811, Mr. Heath, a retired navy sargeon, invented a composition which was far superior to any other in regard to transparency and its natural appearance in the mouth. As, however, the making of this was kept a secret, it had no further effect upon the progress of dentistry than as showing what might be accomplished, and in ultimately bringing a number of able competitors in the field, amongst whom may be mentioned the names of Delepert, Corbett, of Cork, and Hogue, of Edinburgh. The labors of these gentlemen were, however, principally confined to their own practices.

In 1817, Mr. Fonzi invented single mineral teeth, with platina pins for fixing them to the plate; and about the year 1820, Billiard, Desforgues, and others in Paris, introduced teeth of this description into England.

In 1830, Mr. Berrend, of Liverpool, manufactured flat mineral teeth, with platina cramps. These teeth were of a more natural form, and were made in a greater variety of shades of color than had hitherto been obtained. From this time, the manufacture of mineral teeth in this country has continued to advance. Messrs. Ash and Sons were the first to introduce mineral teeth with gold tubes, and were followed, in 1836, by Mr. Lemale. The productions of these old-established firms are too well known to require comment. This branch of manufacture has also of late years been pursued with great success by Messrs. Smale, Taylor, Harnett, Massey, and others.

The foundation having thus been laid, the progress of Dental Science has been extremely rapid during the present century. Many circumstances have combined to produce this result. The anatomy of the teeth, their physiology, and the connections which exist between them and the rest of the body, are better appreciated, although there still remains a wide field of research, which will amply reward the scientific and diligent investigator. A more perfect knowledge of the structure of the teeth has necessarily been accompanied with more correct views of the disease of these organs and their treatment. But, even here, further information is required before we can comprehend in all its bearings the character and history of dental caries. Operations on the teeth are now greatly facilitated by the improved forms and varieties of instruments which have been introduced. The extraction of teeth has been divested of some of its horrors, and we rarely hear of those terrible accidents which formerly so frequently accompanied it. The old key instrument has been superseded by the forceps, and the numerous forms of these instruments at present in use render them available in every operation.

Arriving at these conclusions with respect to Dental Surgery as compared with what it was less than a century ago, two questions will naturally arise: Are dentists more successful in preserving the teeth of their patients than formerly? —and, secondly, to what causes are we to attribute the advancement that has been made? In answer to the first question, I think there are few practitioners who do not feel that at the present time they can preserve teeth which a few years ago would have been condemned to extraction. Formerly, the practitioner, on being consulted, only considered whether the tooth could be immediately stopped, and if not, whether it should not be at once extracted. The plan of temporising, of putting the tooth under a course of treatment so as to bring it into a proper state for plugging was rarely resorted to. The dentist was entirely ignorant of that process of bone deposit by which Nature wards off as far as she can the evil effects of caries. If the patient would not submit to have the tooth extracted, the only alternative was, either to stop it at once or to place some anodyne substance in the cavity of the tooth to relieve the pain. Too often the operator ran the risk, and stopped a tooth which he felt was not in a fit state, and, in consequence, the patient became in a worse state than he was before, and at length his sufferings were so intense that, in spite of his fears, he submitted to the loss of the tooth. It was this kind of injudicious and unskilful treatment which brought dental practice into disrepute, and gave the public less confidence in the curative resources of dentistry.

If we endeavor to ascertain how and by whom this improved state of the profession has been brought about, we must turn to the writings and labors of the scientific practitioner. The example set by Fauchard was soon followed by others; and in France we find the names of several medical men who turned their attention exclusively to dental practice.

In England, the essay of Blake, and the more elaborate treatises of Hunter and Fox, are entitled to the same honorable position. Beardmore, who wrote his work on the teeth in 1768, was one of the first surgeons in this country who confined his attention to dentistry; but Fox bestowed the greatest benefit on the profession by his writings, his works being still worthy of your perusal, and containing much valuable information. Other writers of equal note have since followed in their footsteps, and the names of Bell, Goodsir, Owens, Snell, Koeker, Waite, Robertson, Saunders, Cartwright, Robinson, Salter, Tomes, Richardson, and Hulme, of this country, of Parmley, Goddard, Piggot, Taft, Richardson, Arthur, M'Quillen, Bond, and Chapin Harris, of America; of Jourdain, Bichat, Blandin, Serres, Delabarre, Maury, Audet, and Magitot, in France; of Purkinje in Germany, and Retzius in Sweden, have furnished Dentistry with a rich field of literature, which is principally the production of the last forty years.

During the same period the mechanical department of the profession has undergone changes no less important, so much so, that it may be said to be completely revolutionized. Natural teeth and bone, which were formerly mounted on gold plates, have been almost entirely superseded by mineral teeth, while bone, pieces mounted with natural teeth, have been displaced by vulcanite and mineral teeth; the great change, consisting in indestructible materials, being substituted for those that were perishable. Previous to the introduction of vulcanite, several attempts were made to obtain a material which should be as agreeable to wear as bone, and should, at the same time, be indestructible. With this view Mr. Harrington endeavored to apply tortoise-shell, while in a soft state, to a model of the mouth, and then fitted blocks of mineral teeth to its surface. This gentleman also proposed the use of aluminium for the same purpose, but although his processes displayed much ingenuity, they have not been found to answer the purposes for which they were intended.

Mr. Truman has bestowed much labor upon perfecting gutta percha for dental purposes, and to a certain extent succeeded. In practice, however, several objections have been found to apply to this material; it has since been superseded by vulcanite.

Vulcanite was first employed in mechanical dentistry in 1856, and has since gradually progressed in favor, and is now universally employed where bone was formerly used. This material is so well known, and as I shall again have specially to refer to it in my lectures, it is unnecessary that I should do more than name it on the present occasion.

I have passed over the names of many able practitioners who have enjoyed the confidence of the public; but what I desire to impress upon you most is, that nearly all who have advanced their profession by their writings have received a medical education. It is, in fact, by a combination of scientific knowledge and mechanical skill alone that you can properly qualify yourselves for practice, or hope to rise to eminence in your profession. The time has passed by when mere workmanship will suffice. The public is no longer in that state of ignorance in which they were formerly as regards the teeth: and if you can not answer the questions which an intelligent patient will often put to you, however much he may admire your skill as a workman, he will lose confidence if he find you ignorant of the very organs whose diseases you undertake to treat. "He who is only a mechanic," says Delabarre,* "should not be admitted into

* *Traite de la Partie Mechanique Dentaire.* Paris, 1860.

the Temple of Esculapius, and his duties should be confined to executing under the direction of the surgeon those artificial substitutes which the latter is to apply." On the other hand, however familiar you may be with the anatomy and physiology of the teeth—if you are deficient in mechanical skill your scientific knowledge will not stand you in its stead—for in the words of the writer I have already quoted, "The dentist should have a knowledge of medicine in order that he may avail himself of the laws of physiology in the application of artificial teeth. He should know how to estimate the advantages and disadvantages of the mechanical work which he is required to execute. Sometimes it is an artificial palate, which he must construct and adjust from a perfect knowledge of the forms and uses of the part for which it is substituted, as well as the best means of avoiding the inconvenience it may give rise to. Sometimes by means of an artificial substitute, he endeavors to restore to the wrinkled countenance of the aged, the pleasing appearance of youth—to the voice the tone it had lost, and to the stomach the instruments which nature has provided to aid in the functions of digestion. In order, therefore, to be a good mechanical dentist, the practitioner must have a precise knowledge of two arts essentially distinct—Medicine and Mechanics."

It is, however, to be observed that those who quitted the ranks of the medical profession to practice as dentists were deficient in the mechanical skill which was required in the making of artificial teeth, and hence they had recourse to the assistance of skilled workmen who were originally brought up as jewellers, watch-makers, or in some similar occupation. Out of this combination of educated practitioners and skilled workmen, the present condition of the profession has arisen, and it is to their joint labors we are indebted for the progress which has been made. The defect in this system was that the knowledge necessary to qualify the person for dental practice was not united in one individual, but was divided between the operator and his workmen. It was with a view to remedy this state of things that dental schools were established, and it was hoped that, for the future, those who entered on dental practice would combine in their own persons the knowledge which is necessary to form both a scientific and mechanical practitioner.

The formation of Dental Colleges in America in 1840 was the first attempt to furnish the dental student with the means of properly educating himself for his profession. This example has since been followed in this country, and the College of Dentists was established upon the same principle as had been found to answer in the New World. Simultaneously with the College of Dentists, the Odonotological Society was formed for the promotion of Dental Science. Unfortunately, the members of both societies, although desirous of advancing and reforming the profession, were not agreed as to the means to be employed for that purpose, and hence the antagonism and disunion which at present divide the profession into two parties. The publication of a periodical literature devoted to Dental Science has been another important aid in diffusing knowledge and circulating new discoveries.

All medical and surgical practice must be based upon a knowledge of the form and structure of the body, and this, therefore, is one of the first subjects which will claim your attention. It is not sufficient to know only the parts you are called upon to treat; an acquaintance with their relations to the rest of the body is equally necessary;—in a word, anatomy and physiology must form the foundation of your surgical practice. Your first object should be to make yourself acquainted with the structure of the body and the laws which regulate its functions, and I have no hesitation in saying that, under the guidance of your able Anatomical Lecturer, you will derive pleasure as well as profit from so interesting a study.

It is a source of satisfaction to all who have been engaged in promoting the establishment of this School to know that during the present session that portion of the Anatomical Lectures which come under the name of "Demonstrations," will be taught by one who has been educated in this school—I refer to Mr. Henry, whom many of you have known as a student, and whose industry and perseverance you would do well to emulate.

When you have become acquainted with the organization of the body in health, you will be prepared to appreciate the changes which are produced by disease. Although disease is a deviation from the natural condition of the parts, still even these deviations are subject to certain laws, and hence arise certain principles upon which medical and surgical practice is based. It is these elements of surgical practice which you will learn from a gentleman whose skill and standing in his profession renders him an acquisition to our school.

With regard to Dental Surgery, it is scarcely necessary that I should urge upon you the importance of such a course of lectures in a dental school. In these lectures you learn the theory and principles which are to guide you in practice, but the application of them must be acquired elsewhere. Lectures are valuable as adjuncts to actual experience, but to obtain the latter you must see and examine for yourselves, and above all, you must perform the operations which are required of you in practice. By attendance at the National Dental Hospital, or at similar institutions, you have every opportunity of perfecting yourselves in the treatment of diseases of the teeth, and the various operations which are performed on them. This is a wider and more extended sphere of practice than you can meet in any private surgery, and I am sure that none of us could have listened to the excellent lectures of my colleague, Mr. Hulme, on "Dental Materia Medica," without feeling that the subject was growing in importance, and demanding greater knowledge and higher qualifications than were formerly deemed necessary to fit us for dental practice.

A knowledge of Dental Mechanics can scarcely be deemed less essential on the part of the Dentist than of the anatomical and surgical sciences which have just been alluded to. Here again you can only obtain the required skill by practice in the work-room, but lectures may give you much information as to the best means to be employed, and also of the various plans and suggestions which have been put forward at different times for accomplishing the various operations which are included between the first taking of the impression and the completing a piece of artificial teeth ready for insertion in the mouth. Formerly, and, indeed, to a comparatively recent period, the Dentist imitated the practice of the Egyptians, or of the different trades during the middle ages. He endeavored as far as possible to keep all his proceedings a profound secret, and was jealous of imparting any information to his fellow-practitioners. What he knew he taught his children and apprentices, who initiated into the "art and mystery," were bound to secresy. Happily, we have thrown off some of this illiberal spirit, and there is now a mutual interchange of ideas tending alike to benefit and elevate our Profession.

As the Dentist requires to be familiar with the implements he has to employ, so also should he be acquainted with the qualities and properties of the materials with which he has to work. Chemistry teaches us the nature of the elementary bodies, the compounds which they are capable of forming with each other, and the changes which these combinations produce in their propérties and uses. The various amalgams which have been introduced into dental practice, the alloying of gold, and the different degrees of hardness, elasticity, and fusibility which it acquires from the union of other metals, are the result of chemical research. The changes produced in the teeth by chemical agents, the influences exerted upon the pulp of the tooth by caustics, ac'ds, or anodyne applications, are all of them matters with which you should be familiar, or it is impossible for you to avail yourselves of the various remedial agents which are at your disposal. The science of chemistry has therefore been wisely included in the teachings of this school. It is not only a science which will be of infinite survice to you in the work-room and surgery; in both the mechanical and surgical department of your profession, but it is also one that is calculated to open to you a wide field of knowledge which connects itself with all the arts and sciences of civilized life. Turn where you will, you find chemistry ministering to the wants and luxuries of the people. In the great work-shops of our manufacturing districts there you find it directing the operation of almost every trade. Enter the studo of the artists, and we find chemistry supplying him with the colors that are to embody his ideas, and give them utterance on the canvass.

To these sciences has been added that of Comparative Anatomy, as one which tends to expand the mind, while at the same time it is in perfect accordance with the remainder of your studies. Every man requires a source of mental as well as bodily recreation, but there is no reason that this should not improve as well as recreate.

You have thus provided for you a course of study well adapted to qualify you for the practice of your profession; the actual amount of knowledge which you will derive from it rests with yourselves. A regular attendance upon the lectures is the first thing necessary. Nothing is more discouraging to the lecturer, or more injurious to the student, than a broken and irregular attendance. You lose the connection between the different subjects treated of in the lectures, you find reference made to matters which were mentioned in some lectures when you were not present. The observations of your teachers are consequently obscure; you begin to lose your interest in your subject, and probably cease to attend altogether, or only occasionally, for the purpose of keeping up appearances. Notes of lectures are exceedingly useful, but they should be only notes—that is, the leading points of the lectures; and in order to render them valuable, and to convert them into your own property, if I may so term it, they should be carefully considered, and the parts omitted filled up at your leisure.

In addressing you as students about to commence those studies which are to fit you for your future career in life, I can not hope that any words of mine, however forcible and impressive they might be, can impart to you the great elements of success—namely, industry and a firm determination to labor diligently in the task you have undertaken. These are qualities you ought already to have acquired; but should you be conscious of your deficiency in these matters, let me entreat you to summon all your resolution to overcome defects which will be fatal to your future prospects. You are young, and as yet think little of the value of time; you feel that there are the prospects of many years before, you, and that you can afford to be prodigal of the present. But bear in mind that the present is the seed time of your life, and that as you sow so will you reap hereafter. If the husbandman neglects to till the soil, he can not expect an abundant harvest. After all, it is not for want of knowing what is his duty, or what is necessary to ensure success, that causes the student to fail. We are all possessed with sufficient abilities to acquire the requisite amount of knowledge, but we do not all possess that amount of energy and perseverance, without which the most brilliant talents are but delusive riches.

That a portion of your time should be given to recreation is not only proper, but necessary; but let your alternations of study and relaxation be so arranged and proportioned as to accord with the advice of Bishop Hall. "Recreation," he says, is "adapted to the mind, as whetting is to the scythe, to sharpen the edge of it, which otherwise would grow dull and blunt. He, therefore, that spends his whole time in recreation, is ever whetting, never mowing; his grass may grow and his steed starve. As, contrarily, he that always toils and never recreates is ever mowing, never whetting; laboring much to little purpose; as good no scythe as no edge. Then only doth the work go forward when the scythe is so seasonably and moderately whetted that it may cut, and so cuts that it may have the help of sharpening. I would so interchange, that I neither become dull with work, nor idle and wanton with recreation."

May you so pass the period of your studentship as to lay the foundation of your future prosperity. And may you hereafter look back to the days passed within the walls of this school as some of the most profitable of your lives.

*The Dental Review.*

